# Automated Cerebral Edema Detection using Electroencephalography in Post-Cardiac Arrest Patients

**DOI:** 10.21203/rs.3.rs-8532128/v1

**Published:** 2026-01-19

**Authors:** Rebecca A Stafford, Vedika Srivastava, Aiman Z Altaf, Leigh Ann Mallinger, Allyson L Reinert, Sashank Sai Krishna Madipally, Lindsay R Salvati, Sarah Wahlster, Denise Chen, Emily J Gilmore, David M Greer, Huimin Cheng, Charlene Ong

**Affiliations:** Boston Medical Center; Boston University School of Public Health; Boston University Chobanian & Avedisian School of Medicine; Boston Medical Center; Boston Medical Center; Boston University School of Public Health; Boston University School of Public Health; University of Washington; University of Washington; Yale University; Boston Medical Center; Boston University School of Public Health; Boston University Medical Campus

**Keywords:** Cerebral Edema, Electroencephalography, Transformer

## Abstract

**Aim:**

To develop an electroencephalography (EEG) based machine learning model to identify diffuse cerebral edema in post-cardiac arrest patients and to evaluate its ability to predict edema prior to radiographic detection.

**Methods:**

We performed a retrospective, single-center cohort study of adult patients resuscitated from cardiac arrest (2016-2024) who underwent both neuroimaging and EEG monitoring as part of routine clinical care. Machine learning models using Transformer and Long Short-Term Memory architectures were trained to *detect* diffuse cerebral edema from 4- and 8-hour EEG segments obtained >24 hours after arrest. The best performing detection model was then evaluated for its ability to *predict*diffuse cerebral edema using EEG segments preceding radiographic recognition in patients who ultimately developed edema, compared with matched referents without edema (matched on age, sex, witnessed arrest, and EEG timing). Model performance was assessed using Area Under the Curve (AUC), accuracy, sensitivity, and specificity.

**Results:**

Among 124 patients in the detection model, median age was 53 years, and 74 (59.7%) were male. Sixty-five patients (52.4%) developed diffuse cerebral edema. The best-performing detection model, a Transformer using 4-hour EEG segments, achieved strong performance (AUC 80.0%, accuracy 80.0%, sensitivity 80.0%, specificity 90.0%). In a secondary analysis of 19 patients with diffuse cerebral edema and 19 matched referents, the top-performing prediction model used 8-hour EEG segments (AUC 92.2%, accuracy 90.6%, sensitivity 100%, specificity 88.9%).

**Conclusion:**

Diffuse cerebral edema can be identified in survivors of cardiac arrest using machine learning models applied to routine EEG data. In this study, a Transformer based approach demonstrated superior performance for both detection of established edema and identification of EEG patterns that preceded radiographic recognition than LSTM. With validation in larger and independent cohorts, this strategy may enable earlier recognition of evolving cerebral edema during standard EEG monitoring and support timely interventions to mitigate secondary brain injury.

## Introduction

Diffuse cerebral edema is a well-known complication of hypoxic-ischemic brain injury,^1^ affecting 16–30%^2^ of post-cardiac arrest patients. It is the most frequently identified abnormality on early head computed tomography (CT)^3,4^ and is thought to be a major contributor to morbidity, given its association with poor neurologic and survival outcomes.^4-6^ Diffuse cerebral edema arises from disrupted ionic and osmotic balance following hypoxic-ischemic injury, leading to water accumulation and cellular swelling in the brain.^7,8^ Managing diffuse cerebral edema after cardiac arrest remains challenging because limited data exists to guide targeted treatments for elevated intracranial pressure in this context. As a result, clinicians rely on standard ICP-lowering therapies, yet edema is often recognized only after clinical or radiographic deterioration, limiting opportunities to trial or develop proactive interventions.^5,9-13^

Diffuse cerebral edema is typically identified after alarming clinical signs such as fixed, dilated pupils,^14^ and on imaging by the widespread loss of gray-white differentiation, sulcal effacement, compression of the basal cisterns and ventricles, culminating in transtentorial herniation.^6,15^ However, in its earlier stages, evolving cerebral edema may go unrecognized due to inconsistent imaging intervals^15^ and clinical exam limitations in comatose patients due to both injury severity and confounding effects of sedation,^16^ temperature control,^15^ as well as metabolic derangements.^17^

Electroencephalography (EEG) is non-invasive and widely recommended for seizure detection and prognostication in post-cardiac arrest patients.^18-20^ Diffuse cerebral edema on CT has been associated with an overall suppressed EEG background,^18,21,22^ as well as changes in quantitative EEG features,^16,22^ suggesting that EEG waveform characteristics can be leveraged to automatically detect cerebral edema development at earlier actionable timepoints. While prior work has applied machine learning to EEG data to predict post-discharge outcomes after cardiac arrest,^16,23^ little work has been done to identify or predict in-hospital complications that may impact outcomes, such as diffuse cerebral edema. An automated EEG-based machine learning algorithm could enable non-invasive, real-time monitoring of developing cerebral edema, a capability currently unavailable without advanced, commercially available systems. Standard care depends on intermittent and subjective reviews by epileptologists, often delayed between data acquisition, analysis, and communication to treating teams. Earlier, automated and accurate prediction of diffuse cerebral edema could facilitate the development of targeted therapeutics aimed at mitigating its effects and supporting informed clinical decision-making, as well as enhancing prognostication.

Our study aimed to 1) develop a sensitive and specific machine learning model that classified patients with and without diffuse cerebral edema based on EEG and 2) explored whether such a model could predict diffuse cerebral edema before radiographic recognition.

## Methods

### Setting

We conducted a retrospective single center cohort study at Boston Medical Center, a high- volume urban safety net hospital. During the study period, initially unresponsive cardiac arrest survivors were routinely treated with temperature control (36 to 37 degrees Celsius).^24,25^ Per institutional policy, a non-contrast head CT (NCHCT) was obtained upon arrival, typically within three hours, unless unsafe or infeasible due to patient instability. As clinically indicated, a NCHCT was repeated between hospital days two and three. Brain MRI was obtained when additional information was required for prognostication.

Continuous EEG monitoring was initiated within 24 hours after return of spontaneous circulation (ROSC), or as soon as feasible in patients with impaired consciousness depending on EEG machine availability. EEG was maintained for 24 hours, or longer if seizures, epileptiform discharges, or ongoing coma were present.

### Study population

**Figure 1:**
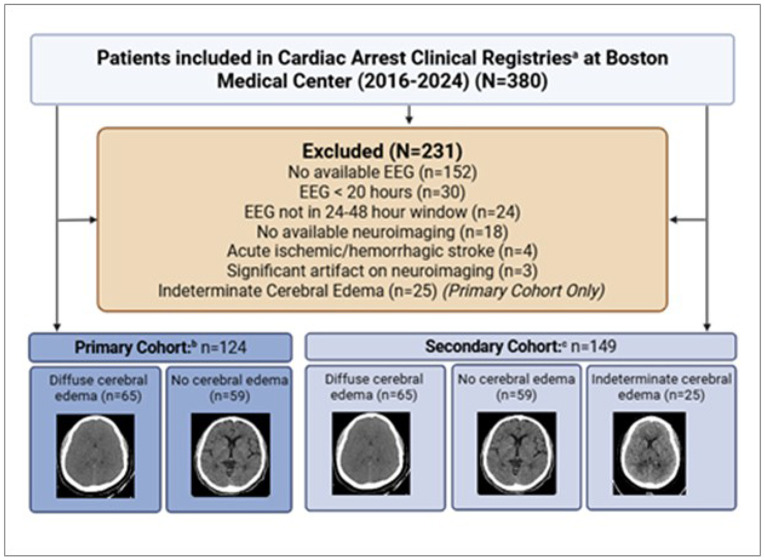
Inclusion and Exclusion Criteria Abb: EEG: electroencephalogram a: Registries included MOCA (N=27); MOCHA (N=253); NEBD (N=84): and Oculography (N=72). Full titles and inclusion and exclusion criteria for the clinical registries listed can be found in the Supplementary Methods. Some patients were included in multiple registries. b:Primary Cohort refers to the 124-patient cohort used for primary detection models. c: Secondary Cohort refers to the 149-patient cohort used to retrain the primary model including indeterminate patients

We included patients admitted between 2016 and 2024 who achieved ROSC after cardiac arrest and were enrolled in IRB-approved cardiac arrest registries. A full list of the clinical registries and their inclusion/exclusion criteria can be found in the Supplemental Methods. Eligible patients were required to have continuous EEG monitoring within the first 24 to 48 hours after arrest and to have at least one neuroimaging study in the first 10 days. Patients were excluded if they had fewer than 20 hours of usable EEG data, technical limitations affecting EEG analysis, or missing neuroimaging. Full exclusion criteria are provided in Fig. 1. The Boston Medical Center IRB (H-37760; H-37113; H-41069; H-40365) approved the study and waived informed consent because data were obtained retrospectively from existing registries and without deviation from standard care.

### Clinical Data Collection

**Figure 2:**
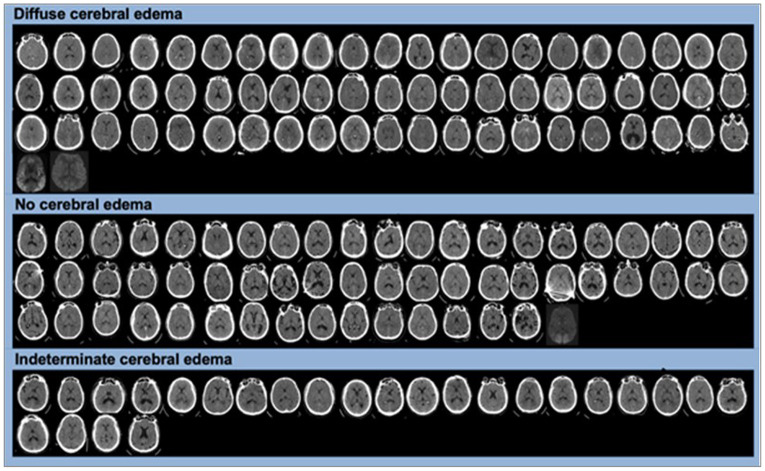
Manual Cerebral Edema Classifications

We extracted clinical data from the electronic health record, including demographics, cardiac arrest characteristics, seizure occurrence, neurological examinations, and outcomes. All neuroimaging obtained within 10 days of arrest was reviewed by a board-certified neurointensivist (CJO) to determine whether patients met diffuse cerebral edema criteria. Diffuse cerebral edema was defined as qualitative assessment of greater than 60% reduction in gray, white matter differentiation or diffusion restriction, sulcal or cisternal effacement, or evidence of herniation (Fig. 2).^6,15^ An independent review by a second neurointensivist (DMG) of 13.4% of cases yielded complete agreement for diffuse cerebral edema. Patients with only focal loss of gray, white differentiation or disagreements between reviewers were classified as indeterminate. Patients were classified as having cerebral edema if criteria were met on any neuroimaging within 10 days of arrest and the onset time was assigned to the first qualifying scan. Continuous EEG recordings, acquired as part of routine clinical care, were exported from the Natus Neuroworks system into a de-identified electronic data format for analysis.^26^

### Electrophysiologic Preprocessing and Augmentation

We selected 4- and 8-hour EEG segments beginning 24 hours after cardiac arrest as model inputs to test performance of both shorter and longer EEG durations at identifying severe cerebral edema. EEG recorded prior to this time was excluded from the primary analysis because early signals are heavily influenced by sedation, targeted temperature management, and other acute interventions and therefore may not reflect intrinsic cerebral physiology. To standardize all recordings, we applied a bipolar montage, using a 0.1–40 Hz bandpass filter to eliminate drift and noise, and resampled the signal at 100 Hz.^27,28^ Artifacts were removed using a validated protocol targeting high-amplitude, low-variance, and rapid-change segments (Supplementary Table 1).^29^ We extracted nine quantitative EEG features capturing different aspects of brain activity: Shannon Entropy, Signal Regularity, Spectral Power (Delta, Theta, Alpha, Beta), Alpha-Delta Ratio, Spike Frequency, and Burst Suppression Ratio.^16^ We augmented training data using controlled noise addition, channel shuffling, and smooth time masking to improve model robustness and generalizability.^30-32^

Using these preprocessed EEG features, we performed two tasks: cerebral edema detection and prediction. The detection task assessed whether EEG patterns could be used to classify patients with and without diffuse cerebral edema. The prediction task evaluated whether the same features could identify cerebral edema, before it was identified radiographically.

### Cerebral Edema Detection

#### Detection Outcomes and Exposures

The primary outcome was the presence of diffuse cerebral edema on radiographic imaging within 10 days of cardiac arrest. The primary exposures were EEG features derived from 4-hour segments of 18 channel EEG beginning 24 hours after cardiac arrest. All patients included in this analysis had more than 20 hours of EEG data, so no missing data imputation was required (Supplementary Table 2). Because the objective of this task was to *detect* cerebral edema from EEG, we did not censor data after radiographic recognition. Additional analyses using 8-, 12-, and 24- hour EEG segments, as well as early EEG (within the first 24 hours after arrest), are described in the Supplementary Methods.

#### Model Training

We trained two machine learning model architectures to detect diffuse cerebral edema. We used a Transformer architecture its effectiveness in capturing long-range dependencies in time series data.^33,34^ For comparison, we trained used a Long Short-Term Memory (LSTM) architecture, which has been applied to EEG data in prior work.^28,35^

Models were initially trained on 84% of the cohort and restricted to patients with either diffuse cerebral edema or no cerebral edema, to ensure the training data reflected clinically confirmed cases of diffuse cerebral edema. The training set was augmented to increase the available data and equally represent both classes of cerebral edema. The remaining 16% were reserved as an independent test set. The test set was balanced with equal numbers of diffuse cerebral edema and non-cerebral edema cases.

Model training was conducted over 150 iterations, each using a different randomly sampled validation subset from the training set to optimize the model. We repeated this process across 30 different train-test splits, ensuring that each patient appeared in only one set per split to avoid data leakage.^36^

#### Model Performance Evaluation

We compared model performance across Transformer and LSTM architectures, with and without data augmentation, using Area Under the Curve (AUC), accuracy, sensitivity, and specificity (threshold 0.5). Performance differences were tested using the Wilcoxon signed rank test, and the best performing model was selected as the final model. Final model parameters for both architectures are shown in Supplementary Tables 3 and 4. An a priori power analysis was conducted: to achieve a sensitivity and specificity of 90 and 95%, respectively, to detect cerebral edema, a minimum of 121 samples was necessary.

EEG feature importance was assessed using Shapley Additive Explanations (SHAP), which estimates the contribution of each input feature to model predictions. SHAP values were averaged across the 30 replications to generate a stable ranking of feature influence.

Sensitivity analyses evaluated model performance in patients with in-hospital versus out-of-hospital cardiac arrest, and using EEG within the first 24 hours after arrest. In a secondary analysis, the best performing model was retrained with indeterminate cases classified as no cerebral edema to assess model performance with label uncertainty.

#### Cerebral Edema Prediction

To explore whether the detection algorithm could identify cerebral edema before it was visible on imaging, we analyzed a subgroup of patients who had at least one hour of EEG data prior to the radiographic detection of edema. Cases were patients who met this outcome and had at least 1-hour of EEG immediately preceding the first radiographic evidence of cerebral edema. Each case was matched to a referent without edema based on age, sex, witnessed arrest status, and time from arrest to EEG using nearest neighbor matching.^37^ Referents were selected from patients who did not develop cerebral edema within 10 days.

#### Prediction Outcomes and Exposures

The outcome remained the presence of diffuse cerebral edema on radiographic imaging within 10 days. For cases, the primary exposures were 4- and 8-hour EEG segments obtained prior to the first radiographic evidence of cerebral edema. This approach ensured that the EEG data reflected brain activity preceding radiographic edema detection, while accounting for variability in the timing of neuroimaging across patients. For matched referents, exposures were drawn from time-matched 4- and 8-hour EEG segments referenced to arrest onset. Missing EEG data were imputed from the average values within the prediction cohort (Supplementary Table 2).

#### Model Training and Performance Evaluation

The best performing detection model was adjusted on this subset to retain representations learned from the full post–cerebral edema dataset while adapting to earlier EEG dynamics present before edema may be present. We allocated 58% of the cohort to training and 42% to testing. As in the detection task, the training set included augmented samples and used 30 train–test splits with 150 iterations per split.

**Figure 3:**
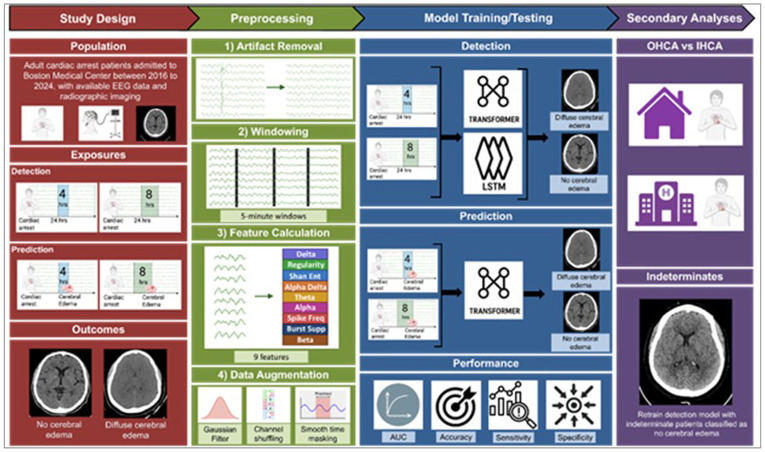
Schematic of cerebral edema detection and prediction using EEG by Transformer and LSTM models Abb: AUC: Area Under Curve; Burst Supp: Burst Suppression; EEG: Electroencephalography; Hrs: Hours; IHCA: In Hospital Cardiac Arrest; LSTM: Long Short-Term Memory; OHCA: Out of Hospital Cardiac Arrest; Shan Ent: Shannon Entropy; Spike Freq: Spike Frequency.

Evaluation metrics matched those used in the detection analysis. Results from the prediction task are exploratory and hypothesis generating. A full study schematic is shown in Fig. 3.

## Results

### Cohort Characteristics

A total of 149 patients met the full eligibility criteria for the study. Twenty-five additional patients were excluded from the primary analysis because cerebral edema was classified as indeterminate (Fig. 1), resulting in 124 patients for the cerebral edema detection cohort. The median age was 53 years (IQR 39.0–60.3), and 74 patients (59.0%) were male. Most arrests were OHCA (101 patients, 81.5%). Diffuse cerebral edema was present in 65 patients (52.4%), and 49 patients (39.5%) survived to discharge. Radiographic features included total loss of gray-white differentiation (n = 45, 36.3%), basilar cistern effacement (n = 46, 37.1%), and evidence of transtentorial herniation (n = 16, 12.9%) (Table 1).

Patients with diffuse cerebral edema were younger and more likely to suffer an unwitnessed OHCA compared to those without edema. Time from arrest to EEG initiation was shorter in the edema group (17.8 hours [IQR 11.3–25.2]) than in the non-edema group (22.1 hours [IQR 15.6–30.0]). Among patients with diffuse cerebral edema, the median time from arrest to initial CT was 1.98 hours [IQR 1.6–3.5] and median time to radiographic edema detection of 14.1 hours [IQR 2.2–49.1]. Of the 124 patients, 33 patients (50.8% of patients with CE) exhibited edema on their initial CT (Table 1). Clinical seizures occurred before edema recognition in 10 patients (15.4%).

Continuous EEG was available before radiographic edema identification in 27 patients, and 19 met criteria for the cerebral edema prediction task and were matched to 19 referents without edema. In the prediction cohort, median age was 55 years [IQR 42.0–59.7] and most were male (n = 32 patients, 84.2%)). Patients with diffuse cerebral edema had a median of 14.2 hours [IQR 4.6–17.4] of EEG before edema was detected on imaging, with 18 patients having a prior CT scan not demonstrating diffuse cerebral edema. The time difference between the end of the EEG analysis window and the radiographic detection of cerebral edema had a median of 1.34 hours [0.29, 1.64] (Supplementary Table 5)

### Cerebral Edema Detection

**Figure 4:**
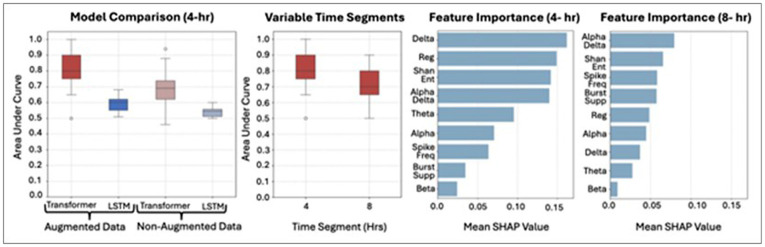
Cerebral Edema Detection Model Performance and Feature Importance Abb: LSTM: Long Short-Term Memory; Hrs: Hours; Reg: Regularity, Shan Ent: Shannon Entropy; Spike Freq: Spike Frequency; Burst Supp: Burst Suppression

The best performing architecture was the Transformer model with data augmentation using 4-hour EEG segments. Across 30 replications, it achieved a median AUC of 80.0% [IQR 75.0–90.0%] and accuracy of 80.0% [IQR 75.0–90.0%], with median sensitivity of 80.0% [IQR 70.0–90.0%] and higher specificity of 90.0% [IQR 80.0–100%]. In contrast, the same model without augmentation performed worse (AUC 69.0% [IQR 62.0–73.8%], accuracy 69.2% [IQR 61.5–76.9%], sensitivity 73.9% [IQR 57.1–87.5%], specificity 61.3% [IQR 50.0–66.7%]). The 4-hour Transformer model without augmentation also significantly outperformed the 4-hour LSTM model across all metrics (Supplementary Table 6). Overall, Transformer models consistently exceeded LSTM performance across all segment lengths and augmentation strategies (Supplementary Fig. 1). Delta power, Regularity, Shannon Entropy, and the Alpha-Delta Ratio were the most influential EEG features in the primary model using 4-hour segments (Fig. 4). Feature importance for other segment lengths is shown in Supplementary Fig. 2.

### Subgroup and Secondary Analysis

The best-performing detection model demonstrated higher sensitivity in patients with OHCA compared with those with IHCA (100% vs 80.0%) (Supplementary Fig. 3). Cohort characteristics for this subgroup are shown in Supplementary Table 7. When patients with indeterminate cerebral edema were included and the model was retrained, performance decreased slightly (median AUC 80.0% [IQR 65.0–85.0%], accuracy 80.0% [IQR 65.0–85.0%]), while median sensitivity (76.9% [IQR 64.4–86.5%]) and specificity (78.9% [IQR 66.7–100%]) remained similar. The 8-hour model performed comparably (Supplementary Fig. 4). In the 4-hour model, the most influential EEG features were Signal Regularity, Shannon Entropy, and Delta Power, whereas Spike Frequency, Shannon Entropy, and Alpha Power were most influential in the 8-hour model. Full results are reported in Supplementary Table 8 and Supplementary Fig. 5.

### Cerebral Edema Prediction

**Figure 5:**
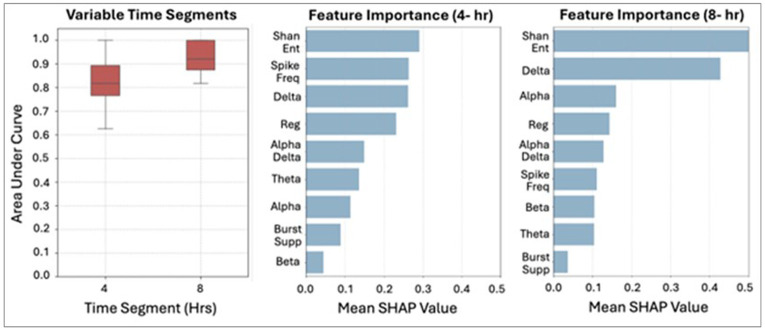
Cerebral Edema Prediction Model Performance and Feature Importance Abb: Reg: Regularity, Shan Ent: Shannon Entropy; Spike Freq: Spike Frequency; Burst Supp: Burst Suppression; Hr: Hour

Twenty-seven patients with diffuse cerebral edema met eligibility for the prediction analysis, and eight were excluded because they had less than 1-hour of EEG prior to edema onset. The final prediction cohort included 19 patients with diffuse cerebral edema (29.2% of all edema cases), each matched to a non–cerebral edema referent (32.2% of non-edema cases). The best adjusted model, trained on 8 hours of pre–cerebral edema EEG, achieved a median AUC of 92.2% [IQR 87.5–100%] and accuracy of 90.6% [IQR 87.5–100%], with median sensitivity of 100% [IQR 87.5–100%] and specificity of 88.9% [IQR 87.5–100%]. Shannon Entropy, Delta Power, and Alpha Power were the most influential EEG features. The fine-tuned 4-hour model performed slightly lower, with a median AUC of 81.7% [IQR 76.7–89.4%], accuracy of 81.2% [IQR 75.0–87.5%], sensitivity of 85.7% [IQR 77.8–100%], and specificity of 83.3% [IQR 73.3–87.5%], with SHAP-based feature importance shown in Fig. 5.

## Discussion

We demonstrated that a machine learning model can detect diffuse cerebral edema using as little as 4 hours of EEG data post-cardiac arrest. We also present preliminary evidence that an automated algorithm can *predict* cerebral edema onset before radiographic recognition.

Machine learning in EEG has growing applications in neuroprognostication and diagnosis, including prediction of neurologic outcomes after cardiac arrest.^16,23,28,38^ Prior work, including our own, leveraged LSTM models to extract prognostic data from EEG and electrooculography (EOG),^16,35,39^ but LSTMs are limited in capturing long-range dependencies in time series data. In contrast, Transformer models leverage self-attention and positional encoding to better understand patterns in time-series data.^33,34^

The best-performing model in our study used a Transformer architecture with data augmentation, which created more variety in the training examples, increasing model flexibility and reducing overfitting. The Transformer's ability to focus on important parts of the data (self-attention) allowed it to make good use of this variety and better recognize key temporal patterns.

We found that 4-hour EEG segments yielded strong model performance in both detecting and predicting diffuse cerebral edema, suggesting that even brief recordings can provide meaningful information. However, 8-hour segments performed best in the prediction task, likely because they captured a broader range of EEG changes that occur as cerebral edema develops, potentially before clinical or radiographic recognition.

The most informative EEG features in both tasks were increased Delta Power, decreased Shannon Entropy, and greater Signal Regularity. Alongside prior evidence demonstrating the utility of these features in neuroprognostication,^16,22,23^ our study provides support for these EEG features, which are indicative of global cortical suppression and seen in burst-suppression physiology, as robust biomarkers of cerebral edema. Subtle differences observed in relative feature importance across different EEG segment durations likely reflect evolving pathophysiology of diffuse cerebral edema and time-dependent^16^ susceptibility to clinical confounders, underscoring the need to consider temporal dynamics when developing real-time prognostic tools.

Subgroup analyses showed that model performance was similar, regardless of arrest location. Model performance decreased when using early EEG segments obtained within the first 24 hours after cardiac arrest, underscoring the importance of considering the timing of EEG acquisition. This reduction is likely due to confounders such as sedation, hemodynamic instability, and acute interventions. Model performance also declined when indeterminate cases were included, suggesting reduced applicability in scenarios where cerebral edema is focal, or evolving.

Continuous EEG is a widely used, guideline-recommended test in cardiac arrest.^40-42^ Our findings support its potential for real-time, non-invasive detection of neurologic worsening. Early EEG-based recognition of cerebral edema prior to current standards, which may be clinical or radiographic depending on patient or institution, could enable timelier interventions compared to the current standard of care, improving patient management and prognostication.

This study has several limitations including the retrospective, single-center design with limited sample size, which may have reduced generalizability and increased the risk of overfitting. Post-hoc EEG artifact removal improved training performance but may have reduced real-world applicability where artifacts are unavoidable. Although data augmentation enhances model robustness, it may have biased features by creating artificial noise not present in native EEG data. Sedation, neuromonitoring interventions, seizures, and circadian dysrhythmia could have influenced EEG data despite starting analysis at 24 hours post-cardiac arrest. Notably, some patients experienced seizures prior to radiographic cerebral edema detection. The relationship between post-arrest seizures and subsequent cerebral edema development remains unclear and requires further research. EEGs may have had fully suppressed backgrounds, however background suppression is non-specific for cerebral edema and may be attributed to overall poor outcome,^22^ or sedative or antiepileptic drugs.^43^

Neuroimaging timing varied by clinical discretion, which may have introduced cerebral edema misclassification. For patients with diffuse cerebral edema on the first post-admission scan, exact edema onset cannot be determined and may have occurred before, during, or after EEG acquisition. Only one case used in the prediction model had diffuse cerebral edema identified on the first scan, suggesting minimal impact on model performance. Limited sample size prevented matching on initial rhythm or time to ROSC, which have known associations with hypoxic-ischemic brain injury. Because clinical “edema detection” is limited either by neuroimaging intervals, or neurologic deterioration (which we did not have access to) EEG segments used in models may reflect ongoing edema that preceded imaging. However, we submit that despite that limitation, identification of edema on EEG remains an important development, as current standards to detect edema rely on late-stage clinical changes or radiographic evidence, obtained at heterogenous intervals, as in our study.

Despite these limitations, we developed the first model that we are aware of that detects and predicts cerebral edema with over 80% AUC in patients with post-cardiac arrest EEG. The implications of real-time identification can enable earlier recognition of neurologic worsening, potential future therapeutic trials, and broader applications across other acute brain injuries that experience cerebral edema. Future studies should validate these findings prospectively in multi-center cohorts with standardized EEG protocols, medication tracking, and imaging criteria, to enhance the model’s clinical utility.

## Conclusion

We developed and validated a Transformer-based model that detects and predicts diffuse cerebral edema in post-cardiac arrest patients using EEG with high AUC, accuracy, sensitivity, and specificity. Transformer architectures consistently outperformed LSTMs, and data augmentation improved performance. These findings suggest that EEG-based machine learning may enable earlier, non-invasive identification of diffuse cerebral edema. Future studies are necessary to validate our findings.

## Supplementary Material

This is a list of supplementary files associated with this preprint. Click to download.

- STROBECENCCJAN2026.docx
- SUPPLEMENTARYMATERIALCE.docx

## Figures and Tables

**Table 1:** Cohort Characteristics

	Total Cohort N = 124	Diffuse Cerebral Edema N = 65 (52.4%)	No Cerebral Edema N = 59 (47.6%)
Demographics			
Age	53 [39, 60.25]	48 [37, 58]	56 [45, 66]
Sex- Male	74 (59.7%)	38 (58.5%)	36 (61.0%)
Race	-	-	-
White	28 (22.6%)	19 (29.2%)	9 (15.3%)
Black	64 (51.6%)	30 (46.2%)	34 (57.6%)
Asian	5 (4.0%)	2 (3.1%)	3 (5.1%)
Other	13 (10.5%)	5 (7.7%)	8 (13.6%)
Unknown/Declined	14 (11.3%)	9 (13.8%)	5 (8.5%)
Hispanic/Latino	17 (13.7%)	8 (12.3%)	9 (15.3%)
Cardiac Arrest Details			
Out of Hospital Arrest	101 (81.5%)	60 (92.3%)	41 (70.7%)
Witnessed Arrest	66 (53.2%)	27 (41.5%)	39 (66.1%)
Initial Rhythm	-	-	-
Asystole	30 (24.2%)	18 (27.7%)	12 (20.3%)
Pulseless Electrical Activity	53 (42.7%)	31 (47.7%)	22 (37.3%)
Ventricular Fibrillation/Tachycardia	19 (15.3%)	5 (7.7%)	14 (23.7%)
Unknown/Other	22 (17.7%)	11 (16.9%)	11 (18.6%)
Hospitalization Details			
Time to Initial Imaging (hr)	2.14 [1.52, 3.50]	1.98 [1.57, 3.47]	2.32 [1.36, 3.70]
Time to EEG (hr)	19.1 [13.5, 27.9]	17.8 [11.3, 25.2]	22.1 [15.6, 30.0]
Time to Radiographic Cerebral Edema Detection (hr)	14.1 [2.2, 49.1]	14.1 [2.2, 49.1]	-
Cerebral Edema diagnosed on first post-admission scan	33 (26.6%)	33 (50.7%)	-
Initial GCS	3 [3, 4]	4 [3, 7.5]	3 [3, 5]
Seizures	46 (37.1%)	23 (35.4%)	23 (39.0%)
Seizures prior to Cerebral Edema	10 (8.1%)	10 (15.4%)	-
Secondary Outcomes			
Downward Herniation	16 (12.9%)	16 (24.6%)	0 (0.0%)
Complete Basilar Cistern Effacement	46 (37.1%)	46 (70.8%)	0 (0.0%)
Loss of Grey-white Differentiation	45 (36.3%)	45 (69.2%)	0 (0.0%)
Discharge Details			
Withdrawal of Life Sustaining Therapy	38 (30.6%)	25 (38.5%)	13 (22.0%)
Brain Death	32 (25.8%)	32 (49.2%)	0 (0.0%)
Discharge modified Rankin Scale (mRS)	-	-	-
mRS 0–3	15 (12.1%)	1 (1.5%)	14 (23.8%)
mRS 4	12 (9.7%)	2 (3.1%)	10 (16.9%)
mRS 5	21 (16.9%)	2 (3.1%)	19 (32.2%)
mRS 6	75 (60.5%)	60 (92.3%)	15 (25.4%)
